# Model order selection for bio-molecular data clustering

**DOI:** 10.1186/1471-2105-8-S2-S7

**Published:** 2007-05-03

**Authors:** Alberto Bertoni, Giorgio Valentini

**Affiliations:** 1 DSI, Dipartimento di Scienze dell' Informazione, Università degli Studi di Milano, Via Comelico 39, Milano, Italy

## Abstract

**Background:**

Cluster analysis has been widely applied for investigating structure in bio-molecular data. A drawback of most clustering algorithms is that they cannot automatically detect the "natural" number of clusters underlying the data, and in many cases we have no enough "a priori" biological knowledge to evaluate both the number of clusters as well as their validity. Recently several methods based on the concept of stability have been proposed to estimate the "optimal" number of clusters, but despite their successful application to the analysis of complex bio-molecular data, the assessment of the statistical significance of the discovered clustering solutions and the detection of multiple structures simultaneously present in high-dimensional bio-molecular data are still major problems.

**Results:**

We propose a stability method based on randomized maps that exploits the high-dimensionality and relatively low cardinality that characterize bio-molecular data, by selecting subsets of randomized linear combinations of the input variables, and by using stability indices based on the overall distribution of similarity measures between multiple pairs of clusterings performed on the randomly projected data. A *χ*^2^-based statistical test is proposed to assess the significance of the clustering solutions and to detect significant and if possible multi-level structures simultaneously present in the data (e.g. hierarchical structures).

**Conclusion:**

The experimental results show that our model order selection methods are competitive with other state-of-the-art stability based algorithms and are able to detect multiple levels of structure underlying both synthetic and gene expression data.

## Background

Unsupervised clustering algorithms play a crucial role in the exploration and identification of structures underlying complex bio-molecular data, ranging from transcriptomics to proteomics and functional genomics [1-4].

Unfortunately, clustering algorithms may find structure in the data, even when no structure is present instead. Moreover, even if we choose an appropriate clustering algorithm for the given data, we need to assess the reliability of the discovered clusters, and to solve the model order selection problem, that is the proper selection of the "natural" number of clusters underlying the data [5,6]. From a machine learning standpoint, this is an intrinsically "ill-posed" problem, since in unsupervised learning we lack an external objective criterion, that is we have not an equivalent of a priori known class label as in supervised learning, and hence the evaluation of the reliability of the discovered classes becomes elusive and difficult. From a biological standpoint, in many cases we have no sufficient biological knowledge to "a priori" evaluate both the number of clusters (e.g. the number of biologically distinct tumor classes), as well as the validity of the discovered clusters (e.g. the reliability of new discovered tumor classes) [7].

To deal with these problems, several methods for assessing the validity of the discovered clusters and to test the existence of biologically meaningful clusters have been proposed (see [8] for a review).

Recently, several methods based on the concept of stability have been proposed to estimate the " optimal" number of clusters in complex bio-molecular data [9-11]. In this conceptual framework multiple clusterings are obtained by introducing perturbations into the original data, and a clustering is considered reliable if it is approximately maintained across multiple perturbations.

Different procedures have been introduced to randomly perturb the data, ranging from bootstrapping techniques [9,12,13], to noise injection into the data [14] or random projections into lower dimensional subspaces [15,16].

In particular, Smolkin and Gosh [17] applied an unsupervised version of the random subspace method [18] to estimate the stability of clustering solutions. By this approach, subsets of features are randomly selected multiple times, and clusterings obtained on the corresponding projected subspaces are compared with the clustering obtained in the original space to assess its stability. Even if this approach gives useful information about the reliability of high-dimensional clusterings, we showed that random subspace projections may induce large distortions in gene expression data, thus obscuring their real structure [15]. Moreover, a major problem with data perturbations obtained through random projections from a higher to a lower dimensional space is the choice of the dimension of the projected subspace.

In this paper we extend the Smolkin and Gosh approach to more general randomized maps from higher to lower-dimensional subspaces, in order to reduce the distortion induced by random projections. Moreover, we introduce a principled method based on the Johnson and Lindenstrauss lemma [19] to properly choose the dimension of the projected subspace. Our proposed stability indices are related to those proposed by Ben-Hur et al. [13]: their stability measures are obtained from the distribution of similarity measures across multiple pairs of clustered data perturbed through resampling techniques. In this work we propose stability indices that depend on the distribution of the similarity measures between pairs of clusterings, but data perturbation is realized through random projections to lower dimensional subspaces, in order to exploit the high-dimensionality of bio-molecular data.

Another major problem related to stability-based methods is to estimate the statistical significance of the structures discovered by clustering algorithms. To face this problem we propose a *χ*^2^-based statistical test that may be applied to any stability method based on the distribution of similarity measures between pairs of clusterings. We experimentally show that by this approach we may discover multiple structures simultaneously present in the data (e.g. hierarchical structures), associating a p-value to the clusterings selected by a given stability-based method for model order selection.

## Methods

In this section we present our approach to stability-based model order selection, considering randomized maps with bounded distortion to perturb the data, stability indices based on the distribution of the clustering similarity measures, and finally we present our *χ*^2^-based test for assessing the significance of the clustering solutions.

### Data perturbations using randomized maps with bounded distortions

A major requirement for clustering algorithms is the reproducibility of their solutions with other data sets drawn from the same source; this is particularly true with bio-molecular data, where the robustness of the solutions is of paramount importance in bio-medical applications. From this standpoint the reliability of a clustering solution is tied to its stability: we may consider reliable a cluster if it is stable, that is if it is maintained across multiple data sets drawn from the same source. In real cases, however, we may dispose only of limited data, and hence we need to introduce multiple " small" perturbations into the original data to simulate multiple "similar" samples from the same underlying unknown distribution. By applying appropriate indices based on similarity measures between clusterings we can then estimate the stability and hence the reliability of the clustering solutions.

We propose to perturb the original data using random projections *μ *: ℝ^*d *^→ ℝ^*d' *^from high *d*-dimensional spaces to lower *d*'-dimensional subspaces. A related approach is presented in [17], where the authors proposed to perturb the data randomly choosing a subset of the original features (*random subspace *projection [18]); the authors did not propose any principled method to choose the dimension of the projected subspace, but a key problem consists in finding a *d' *such that for every pair of data *p*, *q *∈ ℝ^*d*^, the distances between the projections *μ*(*p*) and *μ*(*q*) are approximately preserved with high probability. A natural measure of the approximation is the distortion *dist*_*μ*_:

distμ(p,q)=||μ(p)−μ(q)||2||p−q||2     (1)
 MathType@MTEF@5@5@+=feaafiart1ev1aaatCvAUfKttLearuWrP9MDH5MBPbIqV92AaeXatLxBI9gBaebbnrfifHhDYfgasaacH8akY=wiFfYdH8Gipec8Eeeu0xXdbba9frFj0=OqFfea0dXdd9vqai=hGuQ8kuc9pgc9s8qqaq=dirpe0xb9q8qiLsFr0=vr0=vr0dc8meaabaqaciaacaGaaeqabaqabeGadaaakeaacqWGKbazcqWGPbqAcqWGZbWCcqWG0baDdaWgaaWcbaacciGae8hVd0gabeaakiabcIcaOiabdchaWjabcYcaSiabdghaXjabcMcaPiabg2da9maalaaabaGaeiiFaWNaeiiFaWNae8hVd0MaeiikaGIaemiCaaNaeiykaKIaeyOeI0Iae8hVd0MaeiikaGIaemyCaeNaeiykaKIaeiiFaWNaeiiFaW3aaSbaaSqaaiabikdaYaqabaaakeaacqGG8baFcqGG8baFcqWGWbaCcqGHsislcqWGXbqCcqGG8baFcqGG8baFdaWgaaWcbaGaeGOmaidabeaaaaGccaWLjaGaaCzcamaabmaabaGaeGymaedacaGLOaGaayzkaaaaaa@5AFC@

If *dist*_*μ*_(*p*, *q*) = 1, the distances are preserved; if 1 - *ε *≤ *dist*_*μ*_(*p*, *q*) ≤ 1 + *ε*, we say that an *ε*-*distortion *level is introduced.

In [15] we experimentally showed that random subspace projections used in [17] may introduce large distortions into gene expression data, thus introducing bias into stability indices based on this kind of random projections. For these reasons we propose to apply randomized maps with guaranteed low distortions, according to the *Johnson-Lindenstrauss (JL) lemma *[19], that we restate in the following way: Given a *d*-dimensional data set *D *= {*p*_1_, *p*_2_,...,*p*_*n*_} ⊂ ℝ^*d *^and a distortion level *ε*, randomly choosing a *d*'-dimensional subspace *S *⊂ ℝ^*d*^, with *d' *= *c *log*n*/*ε*^2^, where *c *is a suitable constant, with high probability (say ≥ 0.95) the random projection *μ *:ℝ^*d *^→ *S *verifies 1 - *ε *≤ *dist*_*μ*_(*p*_*i*_, *p*_*j*_) ≤ 1 + *ε *for all *p*_*i *_≠ *p*_*j*_.

In practice, using randomized maps that obey the JL lemma, we may perturb the data introducing only bounded distortions, approximately preserving the metric structure of the original data [15]. Note that the dimension of the projected subspace depends only on the cardinality of the original data and the desired *ε*-distortion, and not from the dimension *d *of the original space.

The embedding exhibited in [19] consists in projections from ℝ^*d *^in random *d'*-dimensional subspaces. Similar results may be obtained by using simpler maps [20,21], represented through random *d' *× *d *matrices R=1/d′(rij)
 MathType@MTEF@5@5@+=feaafiart1ev1aaatCvAUfKttLearuWrP9MDH5MBPbIqV92AaeXatLxBI9gBaebbnrfifHhDYfgasaacH8akY=wiFfYdH8Gipec8Eeeu0xXdbba9frFj0=OqFfea0dXdd9vqai=hGuQ8kuc9pgc9s8qqaq=dirpe0xb9q8qiLsFr0=vr0=vr0dc8meaabaqaciaacaGaaeqabaqabeGadaaakeaacqWGsbGucqGH9aqpcqaIXaqmcqGGVaWldaGcaaqaaiqbdsgaKzaafaaaleqaaOGaeiikaGIaemOCai3aaSbaaSqaaiabdMgaPjabdQgaQbqabaGccqGGPaqkaaa@3844@, where *r*_*ij *_are random variables such that:

*E*[*r*_*ij*_] = 0, *Var*[*r*_*ij*_] = 1

Strictly speaking, these are not projections, but for sake of simplicity, we call random projections even this kind of embeddings. Examples of random projections are the following:

1. *Bernoulli *random projections: represented by *d' *× *d *matrices R=1/d′(rij)
 MathType@MTEF@5@5@+=feaafiart1ev1aaatCvAUfKttLearuWrP9MDH5MBPbIqV92AaeXatLxBI9gBaebbnrfifHhDYfgasaacH8akY=wiFfYdH8Gipec8Eeeu0xXdbba9frFj0=OqFfea0dXdd9vqai=hGuQ8kuc9pgc9s8qqaq=dirpe0xb9q8qiLsFr0=vr0=vr0dc8meaabaqaciaacaGaaeqabaqabeGadaaakeaacqWGsbGucqGH9aqpcqaIXaqmcqGGVaWldaGcaaqaaiqbdsgaKzaafaaaleqaaOGaeiikaGIaemOCai3aaSbaaSqaaiabdMgaPjabdQgaQbqabaGccqGGPaqkaaa@3844@, where *r*_*ij *_are uniformly chosen in {-1, 1}, such that *Prob*(*r*_*ij *_= 1) = *Prob*(*r*_*ij *_= -1) = 1/2 (that is the *r*_*ij *_are *Bernoulli *random variables). In this case the *JL lemma *holds with *c *≃ 4.

2. *Achlioptas *random projections [20]: represented by *d' *× *d *matrices P=1/d′(rij)
 MathType@MTEF@5@5@+=feaafiart1ev1aaatCvAUfKttLearuWrP9MDH5MBPbIqV92AaeXatLxBI9gBaebbnrfifHhDYfgasaacH8akY=wiFfYdH8Gipec8Eeeu0xXdbba9frFj0=OqFfea0dXdd9vqai=hGuQ8kuc9pgc9s8qqaq=dirpe0xb9q8qiLsFr0=vr0=vr0dc8meaabaqaciaacaGaaeqabaqabeGadaaakeaacqWGqbaucqGH9aqpcqaIXaqmcqGGVaWldaGcaaqaaiqbdsgaKzaafaaaleqaaOGaeiikaGIaemOCai3aaSbaaSqaaiabdMgaPjabdQgaQbqabaGccqGGPaqkaaa@3840@, where *r*_*ij *_are chosen in {-3
 MathType@MTEF@5@5@+=feaafiart1ev1aaatCvAUfKttLearuWrP9MDH5MBPbIqV92AaeXatLxBI9gBaebbnrfifHhDYfgasaacH8akY=wiFfYdH8Gipec8Eeeu0xXdbba9frFj0=OqFfea0dXdd9vqai=hGuQ8kuc9pgc9s8qqaq=dirpe0xb9q8qiLsFr0=vr0=vr0dc8meaabaqaciaacaGaaeqabaqabeGadaaakeaadaGcaaqaaiabiodaZaWcbeaaaaa@2DBB@, 0, 3
 MathType@MTEF@5@5@+=feaafiart1ev1aaatCvAUfKttLearuWrP9MDH5MBPbIqV92AaeXatLxBI9gBaebbnrfifHhDYfgasaacH8akY=wiFfYdH8Gipec8Eeeu0xXdbba9frFj0=OqFfea0dXdd9vqai=hGuQ8kuc9pgc9s8qqaq=dirpe0xb9q8qiLsFr0=vr0=vr0dc8meaabaqaciaacaGaaeqabaqabeGadaaakeaadaGcaaqaaiabiodaZaWcbeaaaaa@2DBB@}, such that *Prob*(*r*_*ij *_= 0) = 2/3, *Prob*(*r*_*ij *_= 3
 MathType@MTEF@5@5@+=feaafiart1ev1aaatCvAUfKttLearuWrP9MDH5MBPbIqV92AaeXatLxBI9gBaebbnrfifHhDYfgasaacH8akY=wiFfYdH8Gipec8Eeeu0xXdbba9frFj0=OqFfea0dXdd9vqai=hGuQ8kuc9pgc9s8qqaq=dirpe0xb9q8qiLsFr0=vr0=vr0dc8meaabaqaciaacaGaaeqabaqabeGadaaakeaadaGcaaqaaiabiodaZaWcbeaaaaa@2DBB@) = *Prob*(*r*_*ij *_= -3
 MathType@MTEF@5@5@+=feaafiart1ev1aaatCvAUfKttLearuWrP9MDH5MBPbIqV92AaeXatLxBI9gBaebbnrfifHhDYfgasaacH8akY=wiFfYdH8Gipec8Eeeu0xXdbba9frFj0=OqFfea0dXdd9vqai=hGuQ8kuc9pgc9s8qqaq=dirpe0xb9q8qiLsFr0=vr0=vr0dc8meaabaqaciaacaGaaeqabaqabeGadaaakeaadaGcaaqaaiabiodaZaWcbeaaaaa@2DBB@) = 1/6. In this case also we have *E*[*r*_*ij*_] = 0 and *Var*[*r*_*ij*_] = 1 and the *JL lemma *holds.

3. *Normal *random projections [21,22]: this *JL lemma *compliant randomized map is represented by a *d' *× *d *matrix R=1/d′(rij)
 MathType@MTEF@5@5@+=feaafiart1ev1aaatCvAUfKttLearuWrP9MDH5MBPbIqV92AaeXatLxBI9gBaebbnrfifHhDYfgasaacH8akY=wiFfYdH8Gipec8Eeeu0xXdbba9frFj0=OqFfea0dXdd9vqai=hGuQ8kuc9pgc9s8qqaq=dirpe0xb9q8qiLsFr0=vr0=vr0dc8meaabaqaciaacaGaaeqabaqabeGadaaakeaacqWGsbGucqGH9aqpcqaIXaqmcqGGVaWldaGcaaqaaiqbdsgaKzaafaaaleqaaOGaeiikaGIaemOCai3aaSbaaSqaaiabdMgaPjabdQgaQbqabaGccqGGPaqkaaa@3844@, where *r*_*ij *_are distributed according to a gaussian with 0 mean and unit variance.

4. *Random Subspace *(*RS*) [17,18]: represented by *d' *× *d *matrices R=d/d'(rij)
 MathType@MTEF@5@5@+=feaafiart1ev1aaatCvAUfKttLearuWrP9MDH5MBPbIqV92AaeXatLxBI9gBaebbnrfifHhDYfgasaacH8akY=wiFfYdH8Gipec8Eeeu0xXdbba9frFj0=OqFfea0dXdd9vqai=hGuQ8kuc9pgc9s8qqaq=dirpe0xb9q8qiLsFr0=vr0=vr0dc8meaabaqaciaacaGaaeqabaqabeGadaaakeaacqWGsbGucqGH9aqpdaGcaaqaaiabdsgaKjabc+caViabdsgaKjabcEcaNaWcbeaakiabcIcaOiabdkhaYnaaBaaaleaacqWGPbqAcqWGQbGAaeqaaOGaeiykaKcaaa@396F@, where *r*_*ij *_are uniformly chosen with entries in {0, 1}, and with exactly one 1 per row and at most one 1 per column. Unfortunately, *RS *does not satisfy the *JL lemma*.

Using the above randomized maps (with the exception of *RS *projections), the *JL lemma *guarantees that, with high probability, the "compressed" examples of the data set represented by the matrix *D*_*R *_= *RD *have approximately the same distance (up to a *ε*-distortion level) of the corresponding examples in the original space, represented by the columns of the matrix *D*, as long as *d' *≥ *c *log*n*/*ε*^2^.

We propose a general *MOSRAM *(Model Order Selection by RAndomized Maps) algorithmic scheme, that implements the above ideas about random projection with bounded distortions to generate a set of similarity indices of clusterings obtained by pairs of randomly projected data. The main difference with respect to the method proposed in [13] is that by our approach we perturb the original data using a randomized mapping *μ *: ℝ^*d *^→ ℝ^*d'*^:

#### MOSRAM algorithm

Input:

   *D *: a dataset;

   *k*_*max*_: max number of clusters;

   *m *: number of similarity measures;

   *μ *: a randomized map;

   C
 MathType@MTEF@5@5@+=feaafiart1ev1aaatCvAUfKttLearuWrP9MDH5MBPbIqV92AaeXatLxBI9gBamrtHrhAL1wy0L2yHvtyaeHbnfgDOvwBHrxAJfwnaebbnrfifHhDYfgasaacH8akY=wiFfYdH8Gipec8Eeeu0xXdbba9frFj0=OqFfea0dXdd9vqai=hGuQ8kuc9pgc9s8qqaq=dirpe0xb9q8qiLsFr0=vr0=vr0dc8meaabaqaciaacaGaaeqabaWaaeGaeaaakeaaimaacqWFce=qaaa@3825@: a clustering algorithm;

   *sim *: a clustering similarity measure.

Output:

   *M*(*i*, *k*): a bidimensional list of similarity measures for each *k *(1 ≤ *i *≤ *m*, 2 ≤ *k *≤ *k*_*max*_)

begin

   for *k *:= 2 to *k*_*max*_

      for *i *:= 1 to *m*

      begin

         *proj*_*a *_:= *μ*(*D*)

         *proj*_*b *_:= *μ*(*D*)

         *C*_*a *_:= C
 MathType@MTEF@5@5@+=feaafiart1ev1aaatCvAUfKttLearuWrP9MDH5MBPbIqV92AaeXatLxBI9gBamrtHrhAL1wy0L2yHvtyaeHbnfgDOvwBHrxAJfwnaebbnrfifHhDYfgasaacH8akY=wiFfYdH8Gipec8Eeeu0xXdbba9frFj0=OqFfea0dXdd9vqai=hGuQ8kuc9pgc9s8qqaq=dirpe0xb9q8qiLsFr0=vr0=vr0dc8meaabaqaciaacaGaaeqabaWaaeGaeaaakeaaimaacqWFce=qaaa@3825@(*proj*_*a*_, *k*)

         *C*_*b *_:= C
 MathType@MTEF@5@5@+=feaafiart1ev1aaatCvAUfKttLearuWrP9MDH5MBPbIqV92AaeXatLxBI9gBamrtHrhAL1wy0L2yHvtyaeHbnfgDOvwBHrxAJfwnaebbnrfifHhDYfgasaacH8akY=wiFfYdH8Gipec8Eeeu0xXdbba9frFj0=OqFfea0dXdd9vqai=hGuQ8kuc9pgc9s8qqaq=dirpe0xb9q8qiLsFr0=vr0=vr0dc8meaabaqaciaacaGaaeqabaWaaeGaeaaakeaaimaacqWFce=qaaa@3825@(*proj*_*b*_, *k*)

         *M*(*i*, *k*) := *sim*(*C*_*a*_, *C*_*b*_)

   end

end.

The algorithm computes *m *similarity measures for each desired number of clusters *k*. Every measure is achieved by applying *sim *to the clustering *C*_*a *_and *C*_*b*_, outputs of the clustering algorithm C
 MathType@MTEF@5@5@+=feaafiart1ev1aaatCvAUfKttLearuWrP9MDH5MBPbIqV92AaeXatLxBI9gBamrtHrhAL1wy0L2yHvtyaeHbnfgDOvwBHrxAJfwnaebbnrfifHhDYfgasaacH8akY=wiFfYdH8Gipec8Eeeu0xXdbba9frFj0=OqFfea0dXdd9vqai=hGuQ8kuc9pgc9s8qqaq=dirpe0xb9q8qiLsFr0=vr0=vr0dc8meaabaqaciaacaGaaeqabaWaaeGaeaaakeaaimaacqWFce=qaaa@3825@, having as input *k *and the projected data *proj*_*a *_and *proj*_*b*_. These data are generated through randomized maps *μ*, with a desired distortion level *ε*. It is worth noting that we make no assumptions about the shape of the clusters, and in principle any clustering algorithm C
 MathType@MTEF@5@5@+=feaafiart1ev1aaatCvAUfKttLearuWrP9MDH5MBPbIqV92AaeXatLxBI9gBamrtHrhAL1wy0L2yHvtyaeHbnfgDOvwBHrxAJfwnaebbnrfifHhDYfgasaacH8akY=wiFfYdH8Gipec8Eeeu0xXdbba9frFj0=OqFfea0dXdd9vqai=hGuQ8kuc9pgc9s8qqaq=dirpe0xb9q8qiLsFr0=vr0=vr0dc8meaabaqaciaacaGaaeqabaWaaeGaeaaakeaaimaacqWFce=qaaa@3825@, randomized map *μ*, and clustering similarity measure *sim *may be used (e.g. the Jaccard or the Fowlkes and Mallows coefficients [23]).

### Stability indices based on the distribution of the similarity measures

Using the similarity measures obtained through the *MOSRAM *algorithm, we may compute stability indices to assess the reliability of clustering solutions.

More precisely, let C
 MathType@MTEF@5@5@+=feaafiart1ev1aaatCvAUfKttLearuWrP9MDH5MBPbIqV92AaeXatLxBI9gBamrtHrhAL1wy0L2yHvtyaeHbnfgDOvwBHrxAJfwnaebbnrfifHhDYfgasaacH8akY=wiFfYdH8Gipec8Eeeu0xXdbba9frFj0=OqFfea0dXdd9vqai=hGuQ8kuc9pgc9s8qqaq=dirpe0xb9q8qiLsFr0=vr0=vr0dc8meaabaqaciaacaGaaeqabaWaaeGaeaaakeaaimaacqWFce=qaaa@3825@ be a clustering algorithm, *ρ *a random perturbation procedure (e.g. a resampling or a random projection) and *sim *a suitable similarity measure between two clusterings (e.g. the Fowlkes and Mallows similarity).

We may define the random variable *S*_*k*_, 0 ≤ *S*_*k *_≤ 1:

*S*_*k *_= *sim*(C
 MathType@MTEF@5@5@+=feaafiart1ev1aaatCvAUfKttLearuWrP9MDH5MBPbIqV92AaeXatLxBI9gBamrtHrhAL1wy0L2yHvtyaeHbnfgDOvwBHrxAJfwnaebbnrfifHhDYfgasaacH8akY=wiFfYdH8Gipec8Eeeu0xXdbba9frFj0=OqFfea0dXdd9vqai=hGuQ8kuc9pgc9s8qqaq=dirpe0xb9q8qiLsFr0=vr0=vr0dc8meaabaqaciaacaGaaeqabaWaaeGaeaaakeaaimaacqWFce=qaaa@3825@(*D*_1_, *k*), C
 MathType@MTEF@5@5@+=feaafiart1ev1aaatCvAUfKttLearuWrP9MDH5MBPbIqV92AaeXatLxBI9gBamrtHrhAL1wy0L2yHvtyaeHbnfgDOvwBHrxAJfwnaebbnrfifHhDYfgasaacH8akY=wiFfYdH8Gipec8Eeeu0xXdbba9frFj0=OqFfea0dXdd9vqai=hGuQ8kuc9pgc9s8qqaq=dirpe0xb9q8qiLsFr0=vr0=vr0dc8meaabaqaciaacaGaaeqabaWaaeGaeaaakeaaimaacqWFce=qaaa@3825@(*D*_2_, *k*))     (2)

where *D*_1 _= *ρ*^(1)^(*D*) and *D*_2 _= *ρ*^(2)^(*D*) are obtained through random and independent perturbations of the data set *D*; the intuitive idea is that if *S*_*k *_is concentrated close to 1, the corresponding clustering is stable with respect to a given controlled perturbation and hence it is reliable.

Let *f*_*k*_(*s*) be the density function of *S*_*k *_and *F*_*k*_(*s*) its cumulative distribution function. A parameter of concentration implicitly used in [13] is the integral *g*(*k*) of the cumulative distribution:

g(k)=∫01Fk(s)ds     (3)
 MathType@MTEF@5@5@+=feaafiart1ev1aaatCvAUfKttLearuWrP9MDH5MBPbIqV92AaeXatLxBI9gBaebbnrfifHhDYfgasaacH8akY=wiFfYdH8Gipec8Eeeu0xXdbba9frFj0=OqFfea0dXdd9vqai=hGuQ8kuc9pgc9s8qqaq=dirpe0xb9q8qiLsFr0=vr0=vr0dc8meaabaqaciaacaGaaeqabaqabeGadaaakeaacqWGNbWzcqGGOaakcqWGRbWAcqGGPaqkcqGH9aqpdaWdXaqaaiabdAeagnaaBaaaleaacqWGRbWAaeqaaOGaeiikaGIaem4CamNaeiykaKIaemizaqMaem4CamhaleaacqaIWaamaeaacqaIXaqma0Gaey4kIipakiaaxMaacaWLjaWaaeWaaeaacqaIZaWmaiaawIcacaGLPaaaaaa@4277@

Note that if *S*_*k *_is centered in 1, *g*(*k*) is close to 0, and hence it can be used as a measure of stability. Moreover, the following facts show that *g*(*k*) is strictly related to both the expectation *E*[*S*_*k*_] and the variance *Var*[*S*_*k*_] of the random variable *S*_*k*_:

#### Fact 1

*E*[*S*_*k*_] = 1 - *g*(*k*).

Indeed, integrating by parts:

E[Sk]=∫01sfk(s)ds=∫01sF′k(s)ds=1−∫01Fk(s)ds=1−g(k)     (4)
 MathType@MTEF@5@5@+=feaafiart1ev1aaatCvAUfKttLearuWrP9MDH5MBPbIqV92AaeXatLxBI9gBaebbnrfifHhDYfgasaacH8akY=wiFfYdH8Gipec8Eeeu0xXdbba9frFj0=OqFfea0dXdd9vqai=hGuQ8kuc9pgc9s8qqaq=dirpe0xb9q8qiLsFr0=vr0=vr0dc8meaabaqaciaacaGaaeqabaqabeGadaaakeaacqWGfbqrcqGGBbWwcqWGtbWudaWgaaWcbaGaem4AaSgabeaakiabc2faDjabg2da9maapedabaGaem4CamNaemOzay2aaSbaaSqaaiabdUgaRbqabaGccqGGOaakcqWGZbWCcqGGPaqkcqWGKbazcqWGZbWCcqGH9aqpdaWdXaqaaiabdohaZjqbdAeagzaafaWaaSbaaSqaaiabdUgaRbqabaGccqGGOaakcqWGZbWCcqGGPaqkcqWGKbazcqWGZbWCcqGH9aqpcqaIXaqmcqGHsisldaWdXaqaaiabdAeagnaaBaaaleaacqWGRbWAaeqaaOGaeiikaGIaem4CamNaeiykaKIaemizaqMaem4CamNaeyypa0JaeGymaeJaeyOeI0Iaem4zaCMaeiikaGIaem4AaSMaeiykaKcaleaacqaIWaamaeaacqaIXaqma0Gaey4kIipaaSqaaiabicdaWaqaaiabigdaXaqdcqGHRiI8aaWcbaGaeGimaadabaGaeGymaedaniabgUIiYdGccaWLjaGaaCzcamaabmaabaGaeGinaqdacaGLOaGaayzkaaaaaa@6BEA@

#### Fact 2

*Var*[*S*_*k*_] ≤ *g*(*k*)(1 - *g*(*k*).

Since 0 ≤ *S*_*k *_≤ 1 it follows Sk2
 MathType@MTEF@5@5@+=feaafiart1ev1aaatCvAUfKttLearuWrP9MDH5MBPbIqV92AaeXatLxBI9gBaebbnrfifHhDYfgasaacH8akY=wiFfYdH8Gipec8Eeeu0xXdbba9frFj0=OqFfea0dXdd9vqai=hGuQ8kuc9pgc9s8qqaq=dirpe0xb9q8qiLsFr0=vr0=vr0dc8meaabaqaciaacaGaaeqabaqabeGadaaakeaacqWGtbWudaqhaaWcbaGaem4AaSgabaGaeGOmaidaaaaa@3059@ ≤ *S*_*k*_; therefore, using Fact 1:

*Var*[*S*_*k*_] = *E*[Sk2
 MathType@MTEF@5@5@+=feaafiart1ev1aaatCvAUfKttLearuWrP9MDH5MBPbIqV92AaeXatLxBI9gBaebbnrfifHhDYfgasaacH8akY=wiFfYdH8Gipec8Eeeu0xXdbba9frFj0=OqFfea0dXdd9vqai=hGuQ8kuc9pgc9s8qqaq=dirpe0xb9q8qiLsFr0=vr0=vr0dc8meaabaqaciaacaGaaeqabaqabeGadaaakeaacqWGtbWudaqhaaWcbaGaem4AaSgabaGaeGOmaidaaaaa@3059@] - *E*[*S*_*k*_]^2 ^≤ *E*[*S*_*k*_] - *E*[*S*_*k*_]^2 ^= *g*(*k*)(1 - *g*(*k*))     (5)

In conclusion, *g*(*k*) ≃ 0 then *E*[*S*_*k*_] ≃ 1 and *Var*[*S*_*k*_] = 0, i.e. *S*_*k *_is centered close to 1. As a consequence, *E*[*S*_*k*_] can be used as an index of the reliability of the *k*-clustering: if *E*[*S*_*k*_] ≃ 1, the clustering is stable, if *E*[*S*_*k*_] ≪ 1 the clustering can be considered less reliable.

We can estimate *E*[*S*_*k*_] by means of *m *similarity measures *M*(*i*, *k*)(1 ≤ *i *≤ *m*) computed by the *MOSRAM *algorithm. In fact *E*[*S*_*k*_] may be estimated by the empirical mean *ξ*_*k*_:

ξk=∑i=1mM(i,k)m     (6)
 MathType@MTEF@5@5@+=feaafiart1ev1aaatCvAUfKttLearuWrP9MDH5MBPbIqV92AaeXatLxBI9gBaebbnrfifHhDYfgasaacH8akY=wiFfYdH8Gipec8Eeeu0xXdbba9frFj0=OqFfea0dXdd9vqai=hGuQ8kuc9pgc9s8qqaq=dirpe0xb9q8qiLsFr0=vr0=vr0dc8meaabaqaciaacaGaaeqabaqabeGadaaakeaaiiGacqWF+oaEdaWgaaWcbaGaem4AaSgabeaakiabg2da9maaqahabaWaaSaaaeaacqWGnbqtcqGGOaakcqWGPbqAcqGGSaalcqWGRbWAcqGGPaqkaeaacqWGTbqBaaaaleaacqWGPbqAcqGH9aqpcqaIXaqmaeaacqWGTbqBa0GaeyyeIuoakiaaxMaacaWLjaWaaeWaaeaacqaI2aGnaiaawIcacaGLPaaaaaa@43BA@

### A *χ*^2^-based test for the assessment of the significance of the solutions

In this section we propose a method for automatically finding the " optimal" number of clusters and to detect significant and possibly multi-level structures simultaneously present in the data. First of all, let us consider the vector (*ξ*_2_, *ξ*_3_,...,*ξ*_*H*+1_) (eq. 6) computed by using the output of the *MOSRAM *algorithm. We may perform a sorting of this vector:

(ξ2,ξ3,...,ξH+1)→sort(ξp(1),ξp(2),...,ξp(H))     (7)
 MathType@MTEF@5@5@+=feaafiart1ev1aaatCvAUfKttLearuWrP9MDH5MBPbIqV92AaeXatLxBI9gBaebbnrfifHhDYfgasaacH8akY=wiFfYdH8Gipec8Eeeu0xXdbba9frFj0=OqFfea0dXdd9vqai=hGuQ8kuc9pgc9s8qqaq=dirpe0xb9q8qiLsFr0=vr0=vr0dc8meaabaqaciaacaGaaeqabaqabeGadaaakeaacqGGOaakiiGacqWF+oaEdaWgaaWcbaGaeGOmaidabeaakiabcYcaSiab=57a4naaBaaaleaacqaIZaWmaeqaaOGaeiilaWIaeiOla4IaeiOla4IaeiOla4IaeiilaWIae8NVdG3aaSbaaSqaaiabdIeaijabgUcaRiabigdaXaqabaGccqGGPaqkdaGdKaWcbaGaem4CamNaem4Ba8MaemOCaiNaemiDaqhabeGccaGLsgcacqGGOaakcqWF+oaEdaWgaaWcbaGaemiCaaNaeiikaGIaeGymaeJaeiykaKcabeaakiabcYcaSiab=57a4naaBaaaleaacqWGWbaCcqGGOaakcqaIYaGmcqGGPaqkaeqaaOGaeiilaWIaeiOla4IaeiOla4IaeiOla4IaeiilaWIae8NVdG3aaSbaaSqaaiabdchaWjabcIcaOiabdIeaijabcMcaPaqabaGccqGGPaqkcaWLjaGaaCzcamaabmaabaGaeG4naCdacaGLOaGaayzkaaaaaa@629E@

where *p *is the permutation index such that *ξ*_*p*(1) _≥ *ξ*_*p*(2) _≥ ... ≥ *ξ*_(*H*)_. Roughly speaking, this ordering represents the "most reliable" *p*(1)-clustering down to the least reliable *p*(*H*)-clustering; exploiting this we would establish which are the significant clusterings (if any) discovered in the data.

To this end, for each *k *∈ K
 MathType@MTEF@5@5@+=feaafiart1ev1aaatCvAUfKttLearuWrP9MDH5MBPbIqV92AaeXatLxBI9gBamrtHrhAL1wy0L2yHvtyaeHbnfgDOvwBHrxAJfwnaebbnrfifHhDYfgasaacH8akY=wiFfYdH8Gipec8Eeeu0xXdbba9frFj0=OqFfea0dXdd9vqai=hGuQ8kuc9pgc9s8qqaq=dirpe0xb9q8qiLsFr0=vr0=vr0dc8meaabaqaciaacaGaaeqabaWaaeGaeaaakeaaimaacqWFke=saaa@3835@ = {2, 3,...,*H *+ 1}, let us consider the random variable *S*_*k *_defined in eq. 2, whose expectation is our proposed stability index. For all *k *and for a fixed threshold *t*^*o *^∈ [0, 1] consider the Bernoulli random variable *B*_*k *_= *I*(*S*_*k *_>*t*^*o*^), where *I *is the indicator function: *I*(*P*) = 1 if *P *is True, *I*(*P*) = 0 if *P *is False. The sum Xk=∑j=1mBkj
 MathType@MTEF@5@5@+=feaafiart1ev1aaatCvAUfKttLearuWrP9MDH5MBPbIqV92AaeXatLxBI9gBaebbnrfifHhDYfgasaacH8akY=wiFfYdH8Gipec8Eeeu0xXdbba9frFj0=OqFfea0dXdd9vqai=hGuQ8kuc9pgc9s8qqaq=dirpe0xb9q8qiLsFr0=vr0=vr0dc8meaabaqaciaacaGaaeqabaqabeGadaaakeaacqWGybawdaWgaaWcbaGaem4AaSgabeaakiabg2da9maaqadabaGaemOqai0aa0baaSqaaiabdUgaRbqaaiabdQgaQbaaaeaacqWGQbGAcqGH9aqpcqaIXaqmaeaacqWGTbqBa0GaeyyeIuoaaaa@3B23@ of i.i.d. copies of *B*_*k *_is distributed according to a binomial distribution with parameters *m *and *θ*_*k *_= *Prob*(*I*(*S*_*k *_>*t*^*o*^)).

If we hypothesize that all the binomial populations are independently drawn from the same distribution (i.e. *θ*_*k *_= *θ*, for all *k *∈ K
 MathType@MTEF@5@5@+=feaafiart1ev1aaatCvAUfKttLearuWrP9MDH5MBPbIqV92AaeXatLxBI9gBamrtHrhAL1wy0L2yHvtyaeHbnfgDOvwBHrxAJfwnaebbnrfifHhDYfgasaacH8akY=wiFfYdH8Gipec8Eeeu0xXdbba9frFj0=OqFfea0dXdd9vqai=hGuQ8kuc9pgc9s8qqaq=dirpe0xb9q8qiLsFr0=vr0=vr0dc8meaabaqaciaacaGaaeqabaWaaeGaeaaakeaaimaacqWFke=saaa@3835@), for sufficiently large values of *m *the random variables Xk−mθkmθk(1−θk)
 MathType@MTEF@5@5@+=feaafiart1ev1aaatCvAUfKttLearuWrP9MDH5MBPbIqV92AaeXatLxBI9gBaebbnrfifHhDYfgasaacH8akY=wiFfYdH8Gipec8Eeeu0xXdbba9frFj0=OqFfea0dXdd9vqai=hGuQ8kuc9pgc9s8qqaq=dirpe0xb9q8qiLsFr0=vr0=vr0dc8meaabaqaciaacaGaaeqabaqabeGadaaakeaadaWcaaqaaiabdIfaynaaBaaaleaacqWGRbWAaeqaaOGaeyOeI0IaemyBa0gcciGae8hUde3aaSbaaSqaaiabdUgaRbqabaaakeaadaGcaaqaaiabd2gaTjab=H7aXnaaBaaaleaacqWGRbWAaeqaaOGaeiikaGIaeGymaeJaeyOeI0Iae8hUde3aaSbaaSqaaiabdUgaRbqabaGccqGGPaqkaSqabaaaaaaa@40C5@ are independent and approximately normally distributed. Consider now the random variable:

∑k∈K(Xk−mθ^)2mθ(1−θ)withθ^=∑k∈KXk|K|⋅m     (8)
 MathType@MTEF@5@5@+=feaafiart1ev1aaatCvAUfKttLearuWrP9MDH5MBPbIqV92AaeXatLxBI9gBaebbnrfifHhDYfgasaacH8akY=wiFfYdH8Gipec8Eeeu0xXdbba9frFj0=OqFfea0dXdd9vqai=hGuQ8kuc9pgc9s8qqaq=dirpe0xb9q8qiLsFr0=vr0=vr0dc8meaabaqaciaacaGaaeqabaqabeGadaaakeaafaqabeqadaaabaWaaabuaeaadaWcaaqaaiabcIcaOiabdIfaynaaBaaaleaacqWGRbWAaeqaaOGaeyOeI0IaemyBa0gcciGaf8hUdeNbaKaacqGGPaqkdaahaaWcbeqaaiabikdaYaaaaOqaaiabd2gaTjab=H7aXjabcIcaOiabigdaXiabgkHiTiab=H7aXjabcMcaPaaaaSqaaiabdUgaRjabgIGioprtHrhAL1wy0L2yHvtyaeHbnfgDOvwBHrxAJfwnaGabaiab+Pq8lbqab0GaeyyeIuoaaOqaaiabbEha3jabbMgaPjabbsha0jabbIgaObqaaiqb=H7aXzaajaaaaiabg2da9maalaaabaWaaabeaeaacqWGybawdaWgaaWcbaGaem4AaSgabeaaaeaacqWGRbWAcqGHiiIZcqGFke=saeqaniabggHiLdaakeaacqGG8baFcqGFke=scqGG8baFcqGHflY1cqWGTbqBaaGaaCzcaiaaxMaadaqadaqaaiabiIda4aGaayjkaiaawMcaaaaa@6D7C@

This variable is known to be distributed as a *χ*^2 ^with |K
 MathType@MTEF@5@5@+=feaafiart1ev1aaatCvAUfKttLearuWrP9MDH5MBPbIqV92AaeXatLxBI9gBamrtHrhAL1wy0L2yHvtyaeHbnfgDOvwBHrxAJfwnaebbnrfifHhDYfgasaacH8akY=wiFfYdH8Gipec8Eeeu0xXdbba9frFj0=OqFfea0dXdd9vqai=hGuQ8kuc9pgc9s8qqaq=dirpe0xb9q8qiLsFr0=vr0=vr0dc8meaabaqaciaacaGaaeqabaWaaeGaeaaakeaaimaacqWFke=saaa@3835@| - 1 degrees of freedom, informally because the constraint θ^
 MathType@MTEF@5@5@+=feaafiart1ev1aaatCvAUfKttLearuWrP9MDH5MBPbIqV92AaeXatLxBI9gBaebbnrfifHhDYfgasaacH8akY=wiFfYdH8Gipec8Eeeu0xXdbba9frFj0=OqFfea0dXdd9vqai=hGuQ8kuc9pgc9s8qqaq=dirpe0xb9q8qiLsFr0=vr0=vr0dc8meaabaqaciaacaGaaeqabaqabeGadaaakeaaiiGacuWF4oqCgaqcaaaa@2E79@ between the random variables *X*_*k*_, *k *∈ K
 MathType@MTEF@5@5@+=feaafiart1ev1aaatCvAUfKttLearuWrP9MDH5MBPbIqV92AaeXatLxBI9gBamrtHrhAL1wy0L2yHvtyaeHbnfgDOvwBHrxAJfwnaebbnrfifHhDYfgasaacH8akY=wiFfYdH8Gipec8Eeeu0xXdbba9frFj0=OqFfea0dXdd9vqai=hGuQ8kuc9pgc9s8qqaq=dirpe0xb9q8qiLsFr0=vr0=vr0dc8meaabaqaciaacaGaaeqabaWaaeGaeaaakeaaimaacqWFke=saaa@3835@ introduces a dependence between them, thus leading to a loss of one degree of freedom. By estimating the variance *mθ*(1 - *θ*), with the statistic *m *θ^
 MathType@MTEF@5@5@+=feaafiart1ev1aaatCvAUfKttLearuWrP9MDH5MBPbIqV92AaeXatLxBI9gBaebbnrfifHhDYfgasaacH8akY=wiFfYdH8Gipec8Eeeu0xXdbba9frFj0=OqFfea0dXdd9vqai=hGuQ8kuc9pgc9s8qqaq=dirpe0xb9q8qiLsFr0=vr0=vr0dc8meaabaqaciaacaGaaeqabaqabeGadaaakeaaiiGacuWF4oqCgaqcaaaa@2E79@(1 - θ^
 MathType@MTEF@5@5@+=feaafiart1ev1aaatCvAUfKttLearuWrP9MDH5MBPbIqV92AaeXatLxBI9gBaebbnrfifHhDYfgasaacH8akY=wiFfYdH8Gipec8Eeeu0xXdbba9frFj0=OqFfea0dXdd9vqai=hGuQ8kuc9pgc9s8qqaq=dirpe0xb9q8qiLsFr0=vr0=vr0dc8meaabaqaciaacaGaaeqabaqabeGadaaakeaaiiGacuWF4oqCgaqcaaaa@2E79@), we conclude that the following statistic

Y=∑k∈K(Xk−mθ^)2mθ^(1−θ^)~χ|K|−12     (9)
 MathType@MTEF@5@5@+=feaafiart1ev1aaatCvAUfKttLearuWrP9MDH5MBPbIqV92AaeXatLxBI9gBaebbnrfifHhDYfgasaacH8akY=wiFfYdH8Gipec8Eeeu0xXdbba9frFj0=OqFfea0dXdd9vqai=hGuQ8kuc9pgc9s8qqaq=dirpe0xb9q8qiLsFr0=vr0=vr0dc8meaabaqaciaacaGaaeqabaqabeGadaaakeaacqWGzbqwcqGH9aqpdaaeqbqaamaalaaabaGaeiikaGIaemiwaG1aaSbaaSqaaiabdUgaRbqabaGccqGHsislcqWGTbqBiiGacuWF4oqCgaqcaiabcMcaPmaaCaaaleqabaGaeGOmaidaaaGcbaGaemyBa0Maf8hUdeNbaKaacqGGOaakcqaIXaqmcqGHsislcuWF4oqCgaqcaiabcMcaPaaacqGG+bGFcqWFhpWydaqhaaWcbaGaeiiFaW3enfgDOvwBHrxAJfwnHbqeg0uy0HwzTfgDPnwy1aaceaGae4NcXVKaeiiFaWNaeyOeI0IaeGymaedabaGaeGOmaidaaaqaaiabdUgaRjabgIGiolab+Pq8lbqab0GaeyyeIuoakiaaxMaacaWLjaWaaeWaaeaacqaI5aqoaiaawIcacaGLPaaaaaa@608C@

is approximately distributed according to χ|K|−12
 MathType@MTEF@5@5@+=feaafiart1ev1aaatCvAUfKttLearuWrP9MDH5MBPbIqV92AaeXatLxBI9gBaebbnrfifHhDYfgasaacH8akY=wiFfYdH8Gipec8Eeeu0xXdbba9frFj0=OqFfea0dXdd9vqai=hGuQ8kuc9pgc9s8qqaq=dirpe0xb9q8qiLsFr0=vr0=vr0dc8meaabaqaciaacaGaaeqabaqabeGadaaakeaaiiGacqWFhpWydaqhaaWcbaGaeiiFaW3enfgDOvwBHrxAJfwnHbqeg0uy0HwzTfgDPnwy1aaceaGae4NcXVKaeiiFaWNaeyOeI0IaeGymaedabaGaeGOmaidaaaaa@3FED@ (see, e.g. [24] chapter 12, or [25] chapter 30 for more details).

A realization *x*_*k *_of the random variable *X*_*k *_(and the corresponding realization *y *of *Y*) can be computed by using the output of the *MOSRAM *algorithm:

xk=∑i=1mI(M(i,k)>to)     (10)
 MathType@MTEF@5@5@+=feaafiart1ev1aaatCvAUfKttLearuWrP9MDH5MBPbIqV92AaeXatLxBI9gBaebbnrfifHhDYfgasaacH8akY=wiFfYdH8Gipec8Eeeu0xXdbba9frFj0=OqFfea0dXdd9vqai=hGuQ8kuc9pgc9s8qqaq=dirpe0xb9q8qiLsFr0=vr0=vr0dc8meaabaqaciaacaGaaeqabaqabeGadaaakeaacqWG4baEdaWgaaWcbaGaem4AaSgabeaakiabg2da9maaqahabaGaemysaKKaeiikaGIaemyta0KaeiikaGIaemyAaKMaeiilaWIaem4AaSMaeiykaKIaeyOpa4JaemiDaq3aaWbaaSqabeaacqWGVbWBaaGccqGGPaqkaSqaaiabdMgaPjabg2da9iabigdaXaqaaiabd2gaTbqdcqGHris5aOGaaCzcaiaaxMaadaqadaqaaiabigdaXiabicdaWaGaayjkaiaawMcaaaaa@49BE@

Using *y*, we can test the following alternative hypotheses:

• Ho: all the *θ*_*k *_are equal to *θ*(the considered set of k-clusterings are equally reliable)

• Ha: the *θ*_*k *_are not all equal between them (the considered set of k-clusterings are not equally reliable)

If y≥χα,|K|−12
 MathType@MTEF@5@5@+=feaafiart1ev1aaatCvAUfKttLearuWrP9MDH5MBPbIqV92AaeXatLxBI9gBaebbnrfifHhDYfgasaacH8akY=wiFfYdH8Gipec8Eeeu0xXdbba9frFj0=OqFfea0dXdd9vqai=hGuQ8kuc9pgc9s8qqaq=dirpe0xb9q8qiLsFr0=vr0=vr0dc8meaabaqaciaacaGaaeqabaqabeGadaaakeaacqWG5bqEcqGHLjYSiiGacqWFhpWydaqhaaWcbaGae8xSdeMaeiilaWIaeiiFaW3enfgDOvwBHrxAJfwnHbqeg0uy0HwzTfgDPnwy1aaceaGae4NcXVKaeiiFaWNaeyOeI0IaeGymaedabaGaeGOmaidaaaaa@45A8@ we may reject the null hypothesis at *α *significance level, that is we may conclude that with probability 1 - *α *the considered proportions are different, and hence that at least one k-clustering significantly differs from the others.

Using the above statistical test, we propose an iterative procedure to detect the significant number(s) of clusterings:

1. Consider the ordered vector *ξ *= (*ξ*_*p*(1)_, *ξ*_*p*(2)_,...,*ξ*_*p*(*H*)_)

2. Repeat the *χ*^2^-based test until no significant difference is detected or the only remaining clustering is *p*(1)(the top-ranked one). At each iteration, if a significant difference is detected, remove the bottom-ranked clustering from *ξ*

The output of the proposed procedure is the set of the remaining (top sorted) k-clusterings that correspond to the set of the estimate stable number of clusters (at *α *significance level). Equivalently, following the sorting of *ξ*, we may compute the p-value (probability of committing an error if we reject the null hypothesis) for all the ordered groups of clusterings from the *p*(1)...*p*(*H*) to the the *p*(1), *p*(2) group, each time removing the bottom ranked clustering from the *ξ *vector. Note that if the set of the remaining top-ranked clusterings contains more than one clustering, we may find multiple structures simultaneously present in the data (at *α *significance level).

## Results and discussion

We present experiments with synthetic and gene expression data to show the effectiveness of our approach. At first, using synthetic data, we show that our proposed methods can detect not only the "correct" number of clusters, but also multiple structures underlying the data. Then we apply our *MOSRAM *algorithm to discover the " natural" number of clusters in gene expression data, and we compare the results with other algorithms for model order selection. In our experiments we used the classical *k-means *[26] and *Prediction Around Medoid *(*PAM*) [27] clustering algorithms, and we applied the *Bernoulli, Achlioptas *and *Normal *random projections, but in this section we show only the results obtained with *Bernoulli *projections, since with the other randomized maps we achieved the same results without any significant difference. In all our experiments we set the threshold *t*^*o *^(see Section *"A χ*^2^-*based test for the assessment of the significance of the solutions" *to 0.9. Moreover we applied our proposed *χ*^2^-based procedure to individuate sets of significant *k*-clusterings into the data. The methods and algorithms described in this paper have been implemented in the *mosclust R *package, publicly available at [28].

### Detection of multiple levels of structure in synthetic data

**Figure 1:**
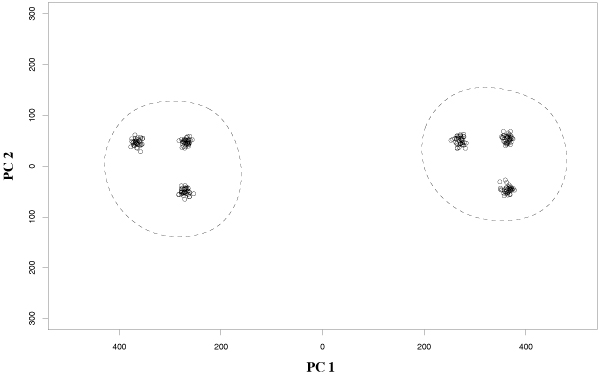
A two-level hierarchical structure with 2 and 6 clusters is revealed by principal components analysis (data projected into the two components with highest variance).

To show the ability of our method to discover multiple structures simultaneously present in the data, we propose an experiment with a 1000-dimensional synthetic multivariate gaussian data set (*sample1*) with relatively low cardinality (60 examples), characterized by a two-level hierarchical structure, highlighted by the projection of the data into the two main principal components (Figure 1): indeed a two-level structure, with respectively 2 and 6 clusters is self-evident in the data.

**Figure 2:**
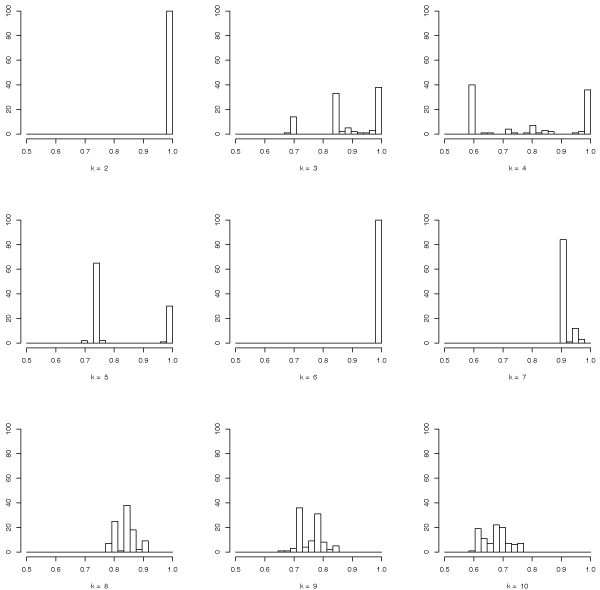
Histograms of the similarity measure distributions for different numbers of clusters.

Two clusterings (using the Prediction Around Medoid algorithm) are detected at 10^-4 ^significance level by applying our *MOSRAM *algorithm and the proposed *χ*^2^-based statistical procedure. In particular we performed 100 pairs of *Bernoulli *projections with a distortion bounded to 1.2 (*ε *= 0.2), yielding to random projections from 1000 to 479-dimensional subspaces. Indeed Table 1 reports the sorted means of the stability measures together with their variance and the corresponding p-values computed according to the proposed *χ*^2^-based statistical test, showing that 2 and 6-clusterings are the best scored, as well as the most significant *k*-clusterings discovered in the data. This situation is depicted in Figure 2, where the histograms of the similarity measures for *k *= 2 and *k *= 6 clusters are tightly concentrated near 1, showing that these clusterings are very stable, while for other values of *k *the similarity measures are spread across multiple values. Note that the p-values of Table 1 (as well as the p-values of Table 2 and 3) refer to the probability of committing an error if we reject the null hypothesis. The clusterings with *p *≥ *α *are considered equally reliable (in this case the null hypothesis cannot be rejected), while the clusterings for which *p *<*α *are considered less reliable (at *α *significance level).

### Experiments with DNA microarray data

To show the effectiveness of our methods with gene expression data we applied *MOSRAM *and the proposed statistical test to *Leukemia *[29] and *Lymphoma *[1] samples. These data sets have been analyzed with other model order selection algorithms previously proposed [10,13,30-32]: at the end of this section we compare the results obtained with the cited methods with our proposed *MOSRAM *algorithm.

#### Leukemia

This well known data set [29] is composed by 72 leukemia samples analyzed with oligonucleotide Affymetrix microarrays. The *Leukemia *data set is composed by a group of 25 acute myeloid leukemia (AML) samples and another group of 47 acute lymphoblastic leukemia (ALL) samples, that can be subdivided into 38 B-Cell and 9 T-Cell subgroups, resulting in a two-level hierarchical structure. We applied the same pre-processing steps performed by the authors of the *Leukemia *study [29], obtaining 3571 genes from the original 7129 gene expression values. We further selected the 100 genes with the highest variance across samples, since low variance genes are unlikely to be informative for the purpose of clustering [10,31]. We analyzed both the 3571-dimensional data and the data restricted to the 100 genes with highest variance, using respectively *Bernoulli *projections with *ε *∈ {0.1, 0.2, 0.3, 0.4} and projections to 80-dimensional subspaces. In both cases the k-means clustering algorithm has been applied.

**Figure 3:**
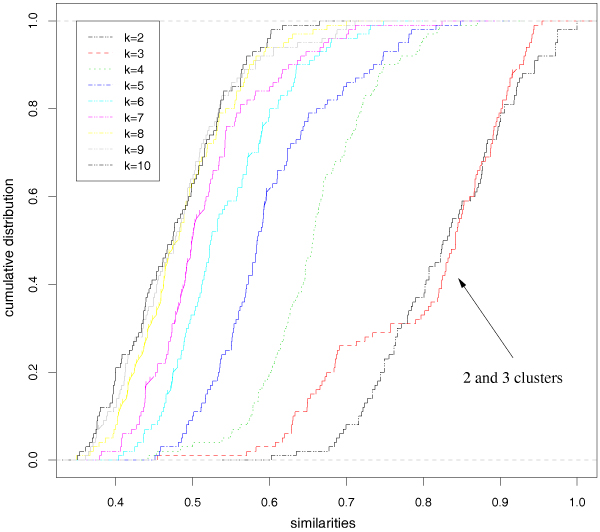
*Leukemia *data set: empirical cumulative distribution functions of the similarity measures for different number of clusters *k*.

Figure 3 summarizes the results using gene expression levels of the genes with highest variance. Table 2 reports the sorted means of the stability measures together with their variance and the corresponding p-values computed according to the proposed *χ*^2^-based statistical test. By these results 2 and 3 clusters are correctly predicted at *α *= 10^-5 ^significance level. Indeed the empirical means of the stability measures (eq. 6) for 2 and 3 clusters are quite similar and the corresponding lines (black and red) of the empirical cumulative distributions (ecdfs) cross several times, while the other ecdfs are clearly apart from them (Figure 3). The same results are approximately obtained also using the 3571-dimensional data with random projections to 428-dimensional subspaces (*ε *= 0.2), but 2 and 3 clusters are predicted at *α *= 10^-13 ^significance level. Similar results are also achieved with *ε *= 0.1 and *ε *= 0.3, while with *ε *= 0.4 the results are less reliable due to the relatively large distortion induced (data not shown). Also using the PAM [27] and hierarchical clustering algorithms with the Ward method [33] we obtained a two-level structure with 2 and 3 clusters at *α *= 10^-5 ^significance level.

#### Lymphoma

Three different lymphoid malignancies are represented in the *Lymphoma *gene expression data set [1]: Diffuse Large B-Cell Lymphoma (DLBCL), Follicular Lymphoma (FL) and Chronic Lymphocytic Leukemia (CLL). The gene expression measurements are obtained with a cDNA microarray specialized for genes related to lymphoid diseases, the Lymphochip, which provides expression levels for 4026 genes [34]. The 62 available samples are subdivided in 42 DLBCL, 11 CLL and 9 FL. We performed pre-processing of the data according to [1], replacing missing values with 0 and then normalizing the data to zero mean and unit variance across genes. As a final step, according to [10], we further selected the 200 genes with highest variance across samples, obtaining a resulting data set with 62 samples and 200 genes. As in the previous experiment, we processed both the high-dimensional original data and the data with the reduced set of high-variance genes, using respectively *Bernoulli *projections with *ε *∈ {0.1, 0.2, 0.3, 0.4} and projections to 160-dimensional subspaces. The k-means clustering algorithm has been applied.

**Figure 4:**
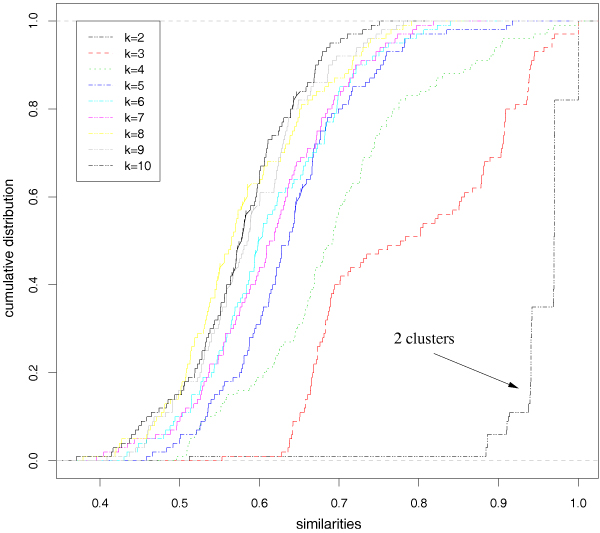
*Lymphoma *data set: empirical cumulative distribution functions of the similarity measures for different number of clusters *k*.

The results with highest variance genes are summarized in Figure 4 and Table 3. The statistical test identifies as significant only the 2-clustering. Indeed, looking at the ecdf of the stability index values (left), the 2-clustering (black) is clearly separated from the others. The 3 (red) and 4-clustering (green) graphs, are quite distinct from the others, as shown also by the corresponding empirical mean of the stability index values (Figure 4), but they are also clearly separated from the 2-clustering curve. Accordingly, our proposed *χ*^2^-based test found a significant difference between the 2-clustering and all the others. Similar results are obtained also with hierarchical clustering and PAM algorithms. Using all the 4026 genes and *Bernoulli *random projections to 413-dimensional (*ε *= 0.2) subspaces with the Ward's hierarchical clustering algorithm our method finds as significant the 3-clustering as well as the 2-clustering. In this case also similar results are obtained with *ε *= 0.1 and *ε *= 0.3. It is worth noting that the subdivision of *Lymphoma *samples in 3 classes (DLBCL, CLL and FL) have been defined on histopathological and morphological basis and it has been shown that this classification does not correspond to the bio-molecular characteristics and to clinical outcome classes of non Hodgkin lymphomas. In particular studies based on the gene expression signatures of the DLBCL patients [1] and on their supervised analysis [35], showed the existence of two subclasses of DLBCLs. Moreover Shipp et al. [36] highlighted that FL patients frequently evolve over time and acquire the clinical features of DLBCLs, and Lange et al. [10] found that a 3-clustering solution groups together FL, CLL and a subgroup of DLBCLs, while another subgroup of DLBCLs sets up another cluster, even if the overall stability of the clustering is lower with respect to the 2-clustering solution. The relationships between FL and subgroups of DLBCL patients are confirmed also by recent studies on the individual stability of the clusters in DLBCL and FL patients [15]. These considerations show also that the stability analysis of patients clusters in DNA microarray analysis are only the first step to discover significant subclasses of pathologies at bio-molecular level, while another necessary step is represented by the bio-medical validation.

#### Comparison with other methods

We compared the results obtained by the *MOSRAM *algorithm with other model order selection methods using the *Leukemia *and *Lymphoma *data sets analyzed in the previous section. In particular we focused our comparison with other state-of-the-art stability-based methods proposed in the literature.

The *Model Explorer *algorithm adopts subsampling techniques to perturb the data (data are randomly drawn without replacement) and applies stability measures based on the empirical distribution of the stability measures [13]. This approach is quite similar to ours but we applied random projections to perturb the data and a statistical test to identify significant numbers of clusters, instead of simply qualitatively looking at the distributions of the stability indices. The *Figure of Merit *measure is based on a resampling approach too, but the stability of the solutions is assessed directly comparing the solution obtained on the full sample with that obtained on the subsamples [32]. We considered also stability-based methods that apply supervised algorithms to assess the quality of the discovered clusterings instead of comparing pairs of perturbed clusterings [10,31]: the main differences between these last approaches are the choice of the supervised predictor and other parameters (no guidance is given in [31], while in [10] a more structured approach is proposed). Finally we considered also a non-stability-based method, the *Gap statistic*, that applies an estimates of the gap between the total sum within-class dissimilarities and a null reference distribution (the uniform distribution on the smallest hyper-rectangle that contains all the data) to assess the "optimal" number of clusters in the data.

Table 4 shows the number of clusters selected by the different methods, as well as their "true" number. The "true" number is estimated according to the a priori biological knowledge about the data [1,29] (see Section *Experiments with DNA microarray data*). The best results achieved with the two gene expression data sets are highlighted with a bounding box. The *MOSRAM *algorithm achieves results competitive with the other state-of-the-art model order selection methods. Indeed *MOSRAM *correctly predicts the "true" number of clusters with the *Leukemia *data set and partially with the *Lymphoma *data set. Note that the 2-clustering prediction with *Lymphoma *may be considered reliable, as outlined in the corresponding experimental section.

These results show that our proposed methods based on randomized maps are well-suited to the characteristics of DNA microarray data: indeed the low cardinality of the examples, the very large number of features (genes) involved in microarray chips, the redundancy of information stored in the spots of microarrays are all characteristics in favour of our approach. On the contrary using bootstrapping techniques to obtain smaller samples from just small samples of patients should induce more randomness in the estimate of cluster stability. A resampling based approach appears to be better suited to evaluate the cluster stability of genes, since significantly larger samples are available in this case [12]. The alternative based on noise injection into the data to obtain multiple instance of perturbed data poses difficult statistical problems for evaluating what kind and which magnitude of noise should be added to the data [17].

All the perturbation-based methods need to properly select a parameter to control the amount of perturbation of the data: resampled-based methods need to select the "optimal" fraction of the data to be subsampled; noise-injection-based methods needs to choice the amount of noise to be introduced; random subspace and random projections-based methods needs to select the proper dimension of the projected data. Anyway, our approach provides a theoretically motivated method to automatically find an "optimal" value for the perturbation parameter, and in our experiments we observed that values of *ε *≤ 0.2 led to reliable results. Moreover our proposed approach provides also a statistical test that may be applied also with other stability-based methods to assess the significance of the discovered solutions.

Despite of the convincing experimental results obtained with stability-based methods there are some drawbacks and open problems associated with these techniques. Indeed, as shown by [8], a given clustering may converge to a suboptimal solution owing to the shape of the data manifold and not to the real structure of the data, thus introducing bias in the stability indices. Moreover in [37] it has been shown that stability based methods based on resampling techniques, when cost-based clustering algorithms are used, may fail to detect the correct number of clusters, if the data are not symmetric. However it is unclear if these results may be extended to other stability-based methods (e.g. to our proposed methods based on random projections) or to other more general classes of clustering algorithms.

## Conclusion

We proposed a stability-based method, based on random projections, for assessing the validity of clusterings discovered in high-dimensional post-genomic data. The reliability of the discovered k-clusterings may be estimated exploiting the distribution of the clustering pairwise similarities, and a *χ*^2^-based statistical test tailored to unsupervised model order selection. In the theoretical framework of randomized maps that satisfy the *JL lemma*, a principled approach to select the dimension of the projected data, and to approximately preserve the structure of the original data is given, thus yielding to the design of reliable stability indices for model order selection in bio-molecular data clusterings.

The *χ*^2^-based statistical test may be applied to any stability method that make use of the distribution of the similarity measures between pairs of clusterings.

Our experimental results with synthetic data and real gene expression data show that our proposed method is able to find significant structures, comprising multiple structures simultaneously present into bio-molecular data.

As an outgoing development, considering that the *χ*^2^-based test assumes that the random variables representing distributions for different number of clusters are normally distributed, we are developing a new distribution-independent approach based on the Bernstein inequality to assess the significance of the discovered k-clusterings.

## Authors' contributions

The authors equally contributed to this paper.

## Figures and Tables

**Table 1:** Samplel: similarity indices. Similarity indices for the synthetic *sample1 *data set for different *k*-clusterings, sorted with respect to their mean values.

**k**	**mean**	**variance**	**p-value**
2	1.0000	0.0000	1.0000
6	1.0000	0.0000	1.0000
7	0.9217	0.0016	0.0000
8	0.8711	0.0033	0.0000
9	0.8132	0.0042	0.0000
5	0.8090	0.0104	0.0000
3	0.8072	0.0157	0.0000
10	0.7715	0.0056	0.0000
4	0.7642	0.0158	0.0000

**Table 2:** Leukemia data set. Stability indices for different *k*-clusterings sorted with respect to their mean values.

**k**	**mean**	**variance**	**p-value**
2	0.8285	0.0077	1.0000
3	0.8060	0.0124	0.7328
4	0.6589	0.0060	2.3279e-06
5	0.6012	0.0073	9.5199e-11
6	0.5424	0.0057	6.3282e-15
7	0.5160	0.0062	0.0000
8	0.4865	0.0050	0.0000
9	0.4819	0.0060	0.0000
10	0.4744	0.0049	0.0000

**Table 3:** Lymphoma data set. Stability indices for different *k*-clusterings sorted with respect to their mean values.

**k**	**mean**	**variance**	**p-value**
2	0.9566	0.0028	1.0000
3	0.7900	0.0149	0.0000
4	0.6963	0.0128	0.0000
5	0.6387	0.0075	0.0000
6	0.6135	0.0082	0.0000
7	0.6129	0.0079	0.0000
9	0.5864	0.0063	0.0000
8	0.5792	0.0079	0.0000
10	0.5744	0.0058	0.0000

**Table 4:** Results comparison. Comparison between different methods for model order selection in gene expression data analysis

Methods	Class. risk (Lange et al. 2004)	Gap statistic (Tibshirani et al. 2001)	Clest (Dudoit and Fridlyand 2002)	Figure of Merit (Levine and Domany 2001)	Model Explorer (BenHur et al 2002)	MOSRAM	"True" number k
Data set							
*Leukemia *(Golub et al. 1999)	k = 3	k = 10	k = 3	k = 2, 8, 19	k = 2	k = 2, 3	k = 2, 3
*Lymphoma *(Alizadeh et al. 2000)	k = 2	k = 4	k = 2	k = 2, 9	k = 2	k = 2	k = 2, (3)
